# Smoking, alcohol consumption and colorectal cancer risk by molecular pathological subtypes and pathways

**DOI:** 10.1038/s41416-020-0803-0

**Published:** 2020-03-30

**Authors:** Efrat L. Amitay, Prudence R. Carr, Lina Jansen, Wilfried Roth, Elizabeth Alwers, Esther Herpel, Matthias Kloor, Hendrik Bläker, Jenny Chang-Claude, Hermann Brenner, Michael Hoffmeister

**Affiliations:** 1 Division of Clinical Epidemiology and Aging Research, German Cancer Research Center (DKFZ), Heidelberg, Germany; 2 Institute of Pathology, University Medical Center Mainz, Mainz, Germany; 3 Institute of Pathology, University Hospital Heidelberg, Heidelberg, Germany; 4 Medical Faculty, Heidelberg University, Heidelberg, Germany; 5 NCT Tissue Bank, National Center for Tumor Diseases (NCT), Heidelberg, Germany; 6 Department of Applied Tumor Biology, Institute of Pathology, University Hospital Heidelberg, Heidelberg, Germany; 7 Institute of Pathology, University hospital Leipzig, Leipzig, Germany; 8 Division of Cancer Epidemiology, German Cancer Research Center (DKFZ), Heidelberg, Germany; 9 Division of Preventive Oncology, German Cancer Research Center (DKFZ) and National Center for Tumor Diseases (NCT), Heidelberg, Germany; 10 German Cancer Consortium (DKTK), German Cancer Research Center (DKFZ), Heidelberg, Germany

**Keywords:** Risk factors, Cancer epidemiology

## Abstract

**Background:**

Smoking and alcohol increase risk for colorectal malignancies. However, colorectal cancer (CRC) is a heterogenic disease and associations with the molecular pathological pathways are unclear.

**Methods:**

This population-based case–control study includes 2444 cases with first-diagnosis CRC and 2475 controls. Tumour tissue was analysed for MSI (microsatellite instability), CIMP (CpG island methylator phenotype), BRAF (B-Raf proto-oncogene serine/threonine kinase gene) and KRAS (Kirsten rat sarcoma viral oncogene homologue gene) mutations. Odds ratios (ORs) and 95% confidence intervals (95% CIs) were estimated for associations between alcohol and smoking and CRC molecular subtypes and pathways.

**Results:**

Current smoking showed higher ORs for MSI-high (OR = 2.79, 95% CI: 1.86–4.18) compared to MSS (OR = 1.41, 1.14–1.75, *p*-heterogeneity (*p*-het) = 0.001), BRAF-mutated (mut) (OR = 2.40, 1.41–4.07) compared to BRAF-wild type (wt) (OR = 1.52, 1.24–1.88, *p*-het = 0.074), KRAS-wt (OR = 1.70, 1.36–2.13) compared to KRAS-mut (OR = 1.26, 0.95–1.68, *p*-het = 0.039) and CIMP-high (OR = 2.01, 1.40–2.88) compared to CIMP-low/negative CRC (OR = 1.50, 1.22–1.85, *p*-het=0.101). Current smoking seemed more strongly associated with sessile serrated pathway (CIMP-high + BRAF-mut; OR = 2.39, 1.27–4.52) than with traditional pathway CRC (MSS + CIMP-low/negative + BRAF-wt; OR = 1.50, 1.16–1.94) and no association was observed with alternate pathway CRC (MSS + CIMP-low/negative + KRAS-wt; OR = 1.08, 0.77–1.43). No heterogeneity was observed in alcohol consumption association by molecular subtypes.

**Conclusions:**

In this large case–control study, smoking was more strongly associated with MSI-high and KRAS-wt CRC and with cases showing features of the sessile serrated pathway. Association patterns were less clear for alcohol consumption.

## Background

Often considered one disease, sporadic colorectal cancer (CRC), accounting for 95% of CRC cases, is a heterogeneous disease arising from different sets of genetic and epigenetic alterations.^1^ The most established underlying molecular pathological subtypes of CRC are characterised by microsatellite instability (MSI) (prevalence 15% in sporadic CRC), CpG island methylator phenotype (CIMP-high, 20%),^2^ B-Raf proto-oncogene serine/threonine kinase gene mutations (BRAF mutations, 10%) and Kirsten rat sarcoma viral oncogene homologue gene mutations (KRAS mutations, 30–50% of sporadic CRC cases). KRAS and BRAF mutations are considered mutually exclusive.^3^ Another important molecular feature is the adenomatous polyposis coli (*APC*) gene, a key tumour suppressor gene mutated in 45% to 81% of sporadic CRC cases.^2^

Smoking is associated with increased risk of CRC.^4^ Moderate to high intake of alcohol was shown to increase CRC risk in a linear dose–response association.^5^ Several studies found smoking was associated with a higher risk of MSI-high,^6–10^ CIMP-high,^8,10,11^ BRAF-mutated (mut)^8,10,11^ and KRAS-wild-type (wt) CRC^12,13^ while others found no differential association by CRC subtypes.^13–16^ In one study, alcohol was found^17^ to be associated with increased risk of MSI-high compared to MSS CRC. In other previous studies, alcohol was not differentially associated with either MSI,^9,18^ CIMP, BRAF^19–21^ or KRAS status.^22^

Since smoking and high alcohol consumption are often correlated, both risk factors were investigated in this study. The aim of this study was to extend current knowledge on the associations of smoking and alcohol consumption with major molecular subtypes and pathways of CRC.

## Methods

### Study population

The DACHS study (Darmkrebs: Chancen der Verhütung durch Screening; CRC: chances for prevention through screening), an ongoing case–control study with follow-up of CRC cases, was initiated in 2003 and has been described in detail previously.^23,24^ In short, cases with a first, histologically confirmed, diagnosis of CRC and randomly selected control participants with no history of CRC, frequency matched to cases by age, sex and county of residence, are recruited in the Rhine-Neckar-Odenwald region in Germany (~2 million inhabitants). The DACHS study was approved by the ethics committees of the Medical Faculty of Heidelberg University and the state medical boards of Baden-Wuerttemberg and Rhineland-Palatinate.

### Data collection

Eligible cases were identified in hospitals within the study region and after giving written informed consent were interviewed by trained interviewers using a standardised questionnaire during hospitalisation after surgery or at home after discharge. The median time between diagnosis and interview was 24 days (interquartile range: 10–224 days). Control participants were randomly selected from population registries and contacted through the study centre to schedule home interviews. Controls with a history of CRC were excluded. Controls opting out of the interview were offered a self-administered short questionnaire. Based on hospital data, ~50% of eligible patients were recruited. The participation rate of eligible controls was 51%.

**Fig. 1:**
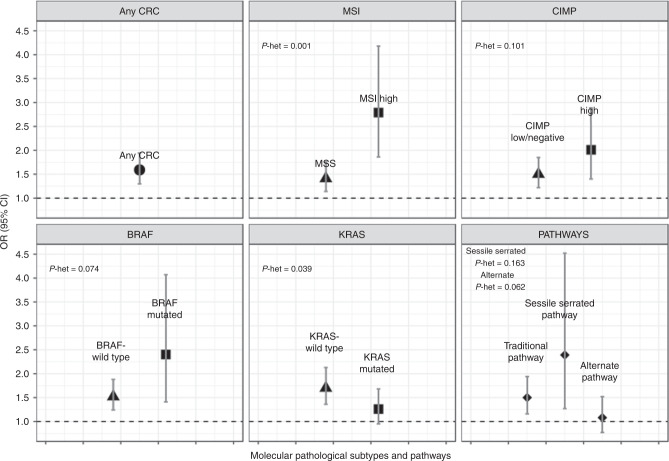
Association of current smoking with CRC risk overall and by molecular pathological subtypes and pathways. Heterogeneity between molecular subtypes was assessed in case-case comparison. For the pathways, heterogeneity was assessed using the traditional pathway as reference.

The current analysis is based on DACHS participants recruited in 2003–2010, as comprehensive molecular tumour analyses of MSI, CIMP, BRAF and KRAS were performed in full for cases joining the study in that period. Participants reporting having Crohn’s disease or ulcerative colitis (*N* = 28) were excluded from the current analysis. Control participants who answered the short questionnaire only (*N* = 658) were also excluded due to missing required information on lifetime alcohol consumption and time of smoking cessation (Supplementary. Fig. 1).

### Assessment of smoking and alcohol consumption

Participants were interviewed regarding smoking history prior to diagnosis (for cases) or interview (controls). Participants were classified as non-smokers if they had never smoked regularly or as former smokers if they had stopped smoking at least 2 years before diagnosis (cases) or interview (controls).

Participants were asked about alcohol consumption in each decade of life from the age of 20 years until diagnosis (cases) or interview (controls). Alcohol consumption was calculated in units of gram ethanol per day. Data were collected on participants’ drinking habits of portions of beer (0.33 l), wine (0.25 l) and liquor (0.02 l). Ethanol content was derived from food composition tables,^25^ assuming an average of 4, 8.6 and 33 g of pure ethanol in 100 ml of beer, wine and liquor, respectively.

### Tumour tissue analyses

Details of tumour tissue analyses of MSI, BRAF, KRAS and CIMP have been reported previously.^26^ In short, formalin-fixed, paraffin-embedded surgical specimens of CRC tumours were collected from cooperating pathology institutes and transferred to the tissue bank at the National Centre for Tumour Diseases (NCT) in Heidelberg. MSI analysis was performed using a mononucleotide marker panel (BAT25, BAT26 and CAT25), which differentiates MSI-high from non-MSI-high tumours with a sensitivity of 98.2% and a specificity of 100%, and with 100% concordance of MSI-high tumours compared with the National Cancer Institute/International Collaborative Group on HNPCC marker panel (BAT25, BAT26, D17S250, D2S123 and D5S346) for the evaluation of MSI in CRC.^27–29^

For KRAS, in about half of the tumour samples, mutation status was determined by a single-stranded conformational polymorphism technique using the same DNA sample, and expression of BRAF V600E was determined by immunohistochemical analyses by two pathologists independently (91% concordance, *κ* 0.59). Discordant cases were discussed to obtain a final evaluation. In the other half of the tumour samples, KRAS mutation status and BRAF mutation status were determined by Sanger sequencing as reported previously.^30^

CIMP was determined after DNA bisulfite conversion as previously described.^31^ CIMP-high and CIMP-low/negative were classified when 3–5 and 0–2 of the investigated loci (MGMT, MLH1, MINT1, MINT2, and MINT31) had a positive methylation status, respectively.

### Statistical analyses

Multinomial logistic regression models were used to estimate adjusted odds ratios (ORs) and 95% confidence intervals (CIs) for the association of smoking and alcohol consumption with CRC risk according to molecular features and pathways. The models were adjusted for covariates known to be associated with CRC risk in all regression analyses: sex, age, body mass index (BMI) 5–14 years before diagnosis (cases) or interview (controls), education level, using non-steroidal anti-inflammatory drugs (NSAIDs) regularly for more than a year (yes, no), history of CRC in a first-degree family member, previous large bowel endoscopy and diabetes. Additionally, average lifetime daily ethanol consumption was included as a covariate in the smoking analyses and smoking (never, former, current) as a covariate in the alcohol consumption analyses. Ever, former and current smoking were compared to never smoking. High alcohol consumption was defined as the fourth quartile of the average daily lifetime gram ethanol consumption among alcohol drinkers (>24.6 g) and was compared in analyses to low/never consumption (≤24.6 g).

In case–control analyses, each of the molecular features or pathways was compared to all study controls. In addition, combinations of single tumour markers approximating the traditional (MSS, CIMP-low/negative, BRAF-wt, KRAS-wt), sessile serrated (CIMP-high, BRAF-mut) and alternate (MSS, CIMP-low/negative, KRAS-mut) pathways to the development of CRC were examined.^32^ To assess heterogeneity in CRC risk between subtypes and pathways, case–case analyses were conducted with the same covariates as in the case–control analyses. All statistical tests were two sided and the significance level (*α*) was <0.05. Analyses were conducted using R version 3.4.4.^33^

## Results

A total of 4919 participants, 2444 cases and 2475 controls, were included in the current analysis. Descriptive statistics for study participants are shown in Table 1. Current smoking was associated with a 59% (OR = 1.59, 95% CI: 1.30–1.94) increased risk of CRC, while former smoking was associated with a 19% (OR = 1.19, 95% CI: 1.03–1.38) increased risk. However, risk was not increased if smoking cessation was more than 20 years ago. More than 29 pack years of smoking were associated with 61% increased CRC risk (OR = 1.61, 95% CI: 1.31–1.99) compared to never smoking. High alcohol consumption (>24.6 g/day) was associated with increased CRC risk (OR = 1.27, 95% CI: 1.08–1.50) (Supplementary Table 1).

**Table 1:** Characteristics of study population.

Variables	Cases (%), *N* = 2444	Controls (%), *N* = 2475	*p* Value^a^
Gender			
Female	1016 (41.6)	974 (40.4)	0.117
Male	1428 (58.4)	1501 (59.6)	
Age (median (range))	70 (30–96)	70 (34–99)	0.474
BMI (kg/m^2^)			
<25	758 (31.5)	928 (37.8)	<0.001
25–30	1168 (48.5)	1179 (48.1)	
>30	480 (20)	346 (14.1)	
School education (years)			
1–8	1672 (68.6)	1474 (59.7)	<0.001
9–10	403 (16.5)	485 (19.6)	
>10	363 (14.9)	511 (20.7)	
Family history of CRC in first-degree relative			
No	2068 (84.8)	2201 (89)	<0.001
Yes	370 (15.2)	271 (11)	
Previous endoscopy			
No	1904 (78.0)	1117 (45.1)	<0.001
Yes	538 (22.0)	1358 (54.9)	
Diabetes			
No	1989 (81.5)	2123 (85.9)	<0.001
Yes	452 (18.5)	348 (14.1)	
Ever regular use of NSAIDs			
Never	1866 (76.7)	1682 (68.4)	<0.001
Yes	568 (23.3)	778 (31.6)	
Physical activity (metabolic equivalents MET-h/week)			
Low	1122 (46.8)	1207 (47.1)	0.136
High	1277 (53.2)	1260 (52.9)	
Smoking			
Never	1134 (46.5)	1257 (50.9)	<0.001
Former	923 (37.9)	945 (38.3)	
Current	380 (15.6)	268 (10.9)	
Avg. lifetime daily alcohol consumption (g ethanol)^b^			
None/low	1872 (76.9)	1996 (79.8)	0.012
High (>24.6 g)	562 (23.1)	473 (20.2)	

Cases and controls were matched by age and sex during recruitment to the study.

*BMI* body mass index (kg/m^2^), *NSAIDs* non-steroidal anti-inflammatory drugs.

^a^Fisher’s exact test.

^b^High alcohol consumption was defined as the fourth quartile of the average daily lifetime gram ethanol consumption among alcohol drinkers (>24.6 g) and was compared in analyses to low/never consumption (≤24.6 g).

### Smoking and CRC risk by molecular pathological subtypes

Current smoking compared to never smoking showed much higher odd-ratios for MSI-high (OR = 2.79, 95% CI: 1.86–4.18) compared to MSS CRC (OR = 1.41, 95% CI: 1.14–1.75, *p*-heterogeneity = 0.001), for BRAF-mut (OR = 2.40, 95% CI: 1.41–4.07) compared to BRAF-wt CRC (OR = 1.52, 95% CI: 1.24–1.88, *p*-het = 0.074), for KRAS-wt (OR = 1.70, 95% CI: 1.36–2.13) compared to KRAS-mut CRC (OR = 1.26, 95% CI: 0.95–1.68, *p*-het=0.039) and for CIMP-high CRC (OR = 2.01, 95% CI: 1.40–2.88) compared to CIMP-low/negative CRC (OR = 1.50, 95% CI: 1.22–1.85, *p*-het = 0.101), although not all differences in association were statistically significant at the *p* < 0.05 level. The results and differences were not as strong for the comparison of ever vs. never smoking (Table 2 and Fig. 1).

**Table 2:** Association of smoking and alcohol consumption with CRC risk by single molecular pathological subtypes.

	Ever regular smoking							Alcohol: g/ethanol/day^a^		
	Never	Ever		Former		Current		≤24.6	>24.6	
	*N* (%)	*N* (%)	OR (95% CI)^b^	*N* (%)	OR (95% CI)^b^	*N* (%)	OR (95% CI)^b^	*N* (%)	*N* (%)	OR (95% CI)^b^
Controls	1257 (50.8)	1217 (49.2)	1	945 (38.3)	1	268 (10.9)	1	1996 (80.8)	473 (19.2)	1
MSI	111 (47.0)	125 (53.0)	1.60 (1.19–2.16)	79 (33.6)	1.26 (0.90–1.76)	45 (19.1)	2.79 (1.86–4.18)	196 (82.7)	41 (17.3)	1.07 (0.72–1.58)
MSS	916 (46.8)	1041 (53.2)	1.24 (1.08–1.43)	756 (38.6)	1.19 (1.03–1.39)	285 (14.6)	1.41 (1.14–1.75)	1486 (76.1)	466 (23.9)	1.32 (1.11–1.57)
*p*-heterogeneity			0.071		0.584		0.001			0.385
BRAF-mut	91 (51.4)	86 (48.6)	1.73 (1.22–2.46)	63 (35.6)	1.56 (1.07–2.28)	23 (13.0)	2.40 (1.41–4.07)	152 (86.4)	24 (13.6)	1.09 (0.61–1.65)
BRAF-wt	955 (46.1)	1118 (53.9)	1.26 (1.10–1.44)	793 (38.3)	1.17 (1.01–1.36)	324 (15.6)	1.52 (1.24–1.88)	1574 (76)	496 (24)	1.30 (1.10–1.54)
*p*-heterogeneity			0.036		0.075		0.074			0.373
KRAS-mut	350 (47.9)	381 (52.1)	1.19 (0.99–1.43)	285 (39.0)	1.17 (0.96–1.43)	96 (13.1)	1.26 (0.95–1.68)	568 (77.9)	161 (22.1)	1.19 (0.94–1.49)
KRAS-wt	690 (45.6)	824 (54.4)	1.34 (1.15–1.55)	577 (38.1)	1.22 (1.04–1.44)	246 (16.3)	1.70 (1.36–2.13)	1147 (75.9)	364 (24.1)	1.30 (1.09–1.56)
*p*-heterogeneity			0.226		0.641		0.039			0.456
CIMP-high	196 (49.2)	202 (50.8)	1.47 (1.15–1.87)	147 (36.9)	1.32 (1.02–1.71)	55 (13.8)	2.01 (1.40–2.88)	323 (81)	76 (19)	1.22 (0.90–1.66)
CIMP-low/neg	930 (46.1)	1088 (53.9)	1.25 (1.09–1.44)	771 (38.2)	1.18 (1.01–1.37)	316 (15.7)	1.50 (1.22–1.85)	1529 (76)	484 (24)	1.28 (1.08–1.52)
*p*-heterogeneity			0.128		0.261		0.101			0.868

^a^Multinomial logistic regression model adjusted for: Sex, age, BMI, education level, history of colorectal cancer in first-degree relative, previous endoscopy, diabetes, ever NSAIDs regular use and average lifetime alcohol consumption/ever regular smoking. Ever/former/current smoking compared to never smoking.

^b^High alcohol consumption was defined as the fourth quartile of the average daily lifetime gram ethanol consumption among alcohol drinkers (>24.6 g) and was compared in analyses to low/never consumption (≤24.6 g).

### Smoking and CRC risk by molecular pathological pathways

Ever and current smoking were significantly associated with higher risk of CRCs that were grouped into the traditional (OR = 1.50, 95% CI: 1.16–1.94) or the serrated pathways (OR = 2.39, 95% CI: 1.27–4.52) (Table 3 and Fig. 1). On the other hand, no association was found between smoking and CRC developing along the alternate pathway, characterised by MSS, CIMP-low/negative and KRAS mutation (OR = 1.08, 95% CI: 0.77–1.52, *p*-het = 0.062).

**Table 3:** Association of smoking and alcohol consumption with CRC risk by molecular pathological pathways.

	Regular smoking							Alcohol: g/ethanol/day^a^		
	Never	Ever		Former		Current		≤24.6	>24.6	
	*N* (%)	*N* (%)	OR (95% CI)^b^	*N* (%)	OR (95% CI)^b^	*N* (%)	OR (95% CI)^b^	*N* (%)	*N* (%)	OR (95% CI)^b^
Controls	1235 (51.1)	1183 (49.4)	1	916 (37.9)	1	267 (11.0)	1	1953 (80.8)	465 (19.2)	1
Traditional pathway	414 (44.5)	517 (55.5)	1.30 (1.09–1.54)	368 (39.5)	1.23 (1.02–1.49)	149 (16.0)	1.50 (1.16–1.94)	680 (73.0)	251 (27.0)	1.57 (1.16–2.11)
Sessile serrated pathway	65 (53.7)	56 (46.3)	1.55 (1.01–2.36)	41 (33.9)	1.34 (0.85–2.13)	15 (12.4)	2.39 (1.27–4.52)	101 (83.5)	20 (16.5)	1.84 (0.75–4.52)
*p*-het traditional vs. sessile serrated			0.340		0.604		0.163			0.850
Alternate pathway	254 (49.4)	260 (50.6)	1.11 (0.9–1.39)	197 (38.3)	1.13 (0.89–1.43)	63 (12.3)	1.08 (0.77–1.52)	397 (77.2)	117 (22.8)	1.15 (0.80–1.66)
*p*-het traditional vs. alternate			0.171		0.425		0.062			0.243

^a^Logistic regression model adjusted for: Sex, age, BMI, education level, history of colorectal cancer in first-degree relative, previous endoscopy, diabetes, ever NSAIDs regular use and average lifetime alcohol consumption/ever regular smoking. Ever/former/current smoking compared to never smoking. High alcohol intake compared to low/none. Traditional pathway: MSS, CIMP-low/negative, BRAF-wt and KRAS-wt; sessile serrated pathway: CIMP-high, BRAF-mut; alternate pathway: MSS, CIMP-low/negative, KRAS-mut.

^b^High alcohol consumption was defined as the third quartile of the average daily lifetime gram ethanol consumption among alcohol drinkers (>24.6 g) and was compared in analyses to low/never consumption (≤24.6 g).

### Alcohol consumption and CRC risk by molecular pathological subtypes and pathways

**Fig. 2:**
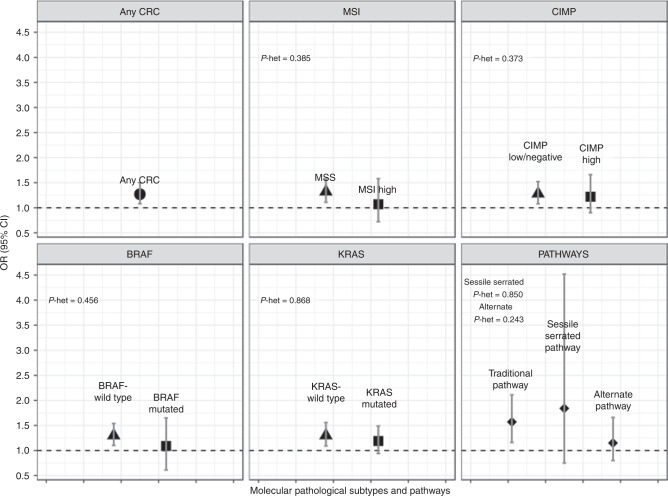
Association of high alcohol consumption with CRC risk overall and by molecular pathological subtypes and pathways. Heterogeneity between molecular subtypes was assessed in case-case comparison. For the pathways, heterogeneity was assessed using the traditional pathway as reference.

Average lifetime daily consumption of more than 24.6 g ethanol was associated with around 30% increased CRC risk of the non-aberrant, more frequent subtypes (MSS, BRAF-wt, KRAS-wt, CIMP-low/neg) and not with the aberrant subtypes (MSI, BRAF-mut, CIMP-high), but no statistically significant differences were observed in heterogeneity testing between subtypes in this study. The strength of the associations of high alcohol consumption with risk of traditional pathway CRC and the direction of association of the serrated pathway CRC were comparable to that of current smoking, but heterogeneity was not statistically significant (Tables 2 and 3 and Fig. 2).

### Joint effects of smoking and alcohol

No interaction was found between high lifetime average daily alcohol consumption and ever smoking (*p*-interaction = 0.519) in the association with CRC risk, and no major differences were found when analysing alcohol consumption (high vs. low/none) stratified by smoking status (ever/never) and CRC risk for the different molecular subtypes or pathways (data not shown).

## Discussion

This large population-based case–control study aimed to examine the association between smoking and alcohol consumption and CRC risk by CRC subtypes and pathways characterised by MSI, BRAF mutation, KRAS mutation and CIMP status. Ever and current smoking were associated with higher risk for CRC, especially with MSI-high, BRAF-mut, KRAS-wt and CIMP-high CRC. Smoking was also associated with higher risk of cancers developing via the traditional or the serrated pathways. High alcohol consumption was not differently associated with single CRC subtypes or pathways, but associations with molecular pathways still seemed similar to those of current smoking.

Our findings are generally in agreement with former studies. Smoking was associated with higher MSI-high compared to MSS CRC risk in five previous studies^6–10^ and in a meta-analysis published in 2018.^34^ Stronger associations were also previously reported for BRAF-mut compared to BRAF-wt CRC,^8,10,11^ for KRAS-wt compared to KRAS-mut CRC^12–14^ and for CIMP-high compared to CIMP-low/negative CRC.^10,11^ Smoking has also been found to be associated with the serrated-polyps pathway, defined by CIMP-high and BRAF-mut status.^35–37^ Further, in accordance with previous studies, no major or statistically significant differences were found in the associations between alcohol consumption and CRC risk by molecular pathological subtypes,^9,17–19,22^ although the observed associations with CRC pathways pointed to potential differences in our study.

Although several possible biological mechanisms were proposed,^10,38^ the way smoking increases the risk of MSI-high CRC is still not established. Our results also support a strong link between smoking and BRAF mutation, which is regarded as the initiating event in sessile serrated adenomas, followed by methylation of key tumour suppressor genes, which would also be supported by the stronger association with CIMP-high CRC and the sessile serrated pathway.^32,39^

Molecular pathological epidemiology (MPE) focuses on heterogenic aetiology of CRC based on molecular tumour features. While studies linking smoking with CRC risk found an overall increase of around 26% in risk,^40,41^ our study provides more specific risk estimates by known CRC subtypes and pathways. This distinction can help provide more evidence for the causal relationship and its mechanisms between smoking and CRC risk.

The large size of the study, its population-based design, the comprehensive assessment of smoking, alcohol consumption and other lifestyle, medical and family history factors, and the analysis of multiple major molecular tumour tissue markers, are notable strengths of this study. To the best of our knowledge, this is the first study examining the effect of both smoking and alcohol consumption on CRC risk by major molecular pathological tumour features and pathways of CRC. This study adds to the limited knowledge about the potential smoking-related increase in CRC risk according to molecular features, potentially linking smoking differentially to specific molecular pathways.

The study also has limitations. MPE is a relatively new field of research, thus analyses are often exploratory and need confirmation from other studies.^1^ Not all patients with available tumour tissue samples could be included in the subtype analyses: MSI status was available for 90% of cases, BRAF for 92%, KRAS for 92% and CIMP for 99% of cases. Another limitation is that some analysed subgroups of cases were small, which lowered the ability of the study to reach significant results, particularly when multiple tumour features were combined. As this is an observational study, based on self-reports during standardised interviews, smoking, alcohol and other relevant factors may be subject to information bias.

In summary, based on results from this large population-based case–control study, smoking, and in particular current smoking, showed the strongest association with increased risk of molecular subtypes of CRC MSI-high and KRAS-wt and with CRC showing features of the sessile serrated pathway. No major differences were observed for the association of alcohol with subtypes, but potential differences according to pathways should be investigated in future studies. More large studies with tumour marker combinations are needed to confirm these results for a better characterisation of the carcinogenic mechanisms underlying these associations.

## Supplementary information

Supplements

## Data Availability

The data used in the current analyses are not publicly available due to legal privacy restrictions.
