# Supplementary material for: Smoking, alcohol consumption and colorectal cancer risk by molecular pathological subtypes and pathways
Source: Br J Cancer. 2020 Mar 30;122(11):1604–10. doi: 10.1038/s41416-020-0803-0 (PMC7250912; doi:10.1038/s41416-020-0803-0)
Supplement: Supplementary file 1 — Supplements [file 41416_2020_803_MOESM1_ESM.docx]

**
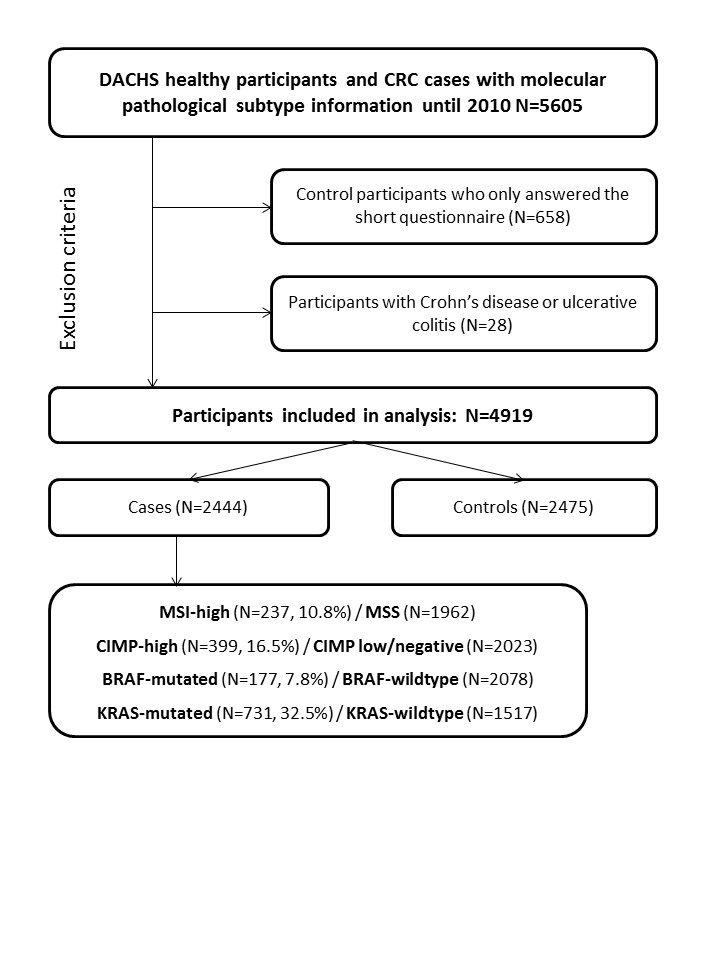
**

**Supplementary figure A** Study population flow diagram

**Supplementary Table A:** Association between smoking and alcohol consumption and colorectal cancer

| ***Variable*** | ***Categories*** | ***Cases***  ***N=2444 (%)*** | ***Controls***  ***N=2475 (%)*** | **Cases vs Controls**  **OR (95%CI)^*^** | ***P - value*** |
| --- | --- | --- | --- | --- | --- |
| **Ever regular smoking** | Non smokers | 1134 (46.5) | 1257 (50.8) | Reference |  |
|  | Yes | 1304 (53.5) | 1217 (49.2) | 1.29 (1.13 to 1.47) | <0.001 |
|  | Former | 923 (37.9) | 945 (38.3) | 1.19 (1.03 to 1.38) | 0.016 |
|  | Current | 380 (15.6) | 268 (10.9) | 1.59 (1.30 to 1.94) | <0.001 |
|  |  |  |  |  |  |
| **Years since smoking secession^+^** | Non smokers | 1134 (55.2) | 1257 (57.1) | Reference |  |
|  | ≤ 20 | 377 (18.3) | 316 (14.4) | 1.42 (1.17 to 1.72) | <0.001 |
|  | > 20 | 544 (26.5) | 628 (28.5) | 1.07 (0.90 to 1.26) | 0.437 |
|  |  |  |  |  |  |
| **Pack-years quartiles^++^** | Non smokers | 1134 (47.5) | 1257 (52.3) | Reference |  |
|  | Q1 (1-6) | 273 (11.4) | 301 (12.5) | 1.11 (0.91, 1.36) | 0.305 |
|  | Q2 (7-15) | 316 (13.2) | 289 (12.0) | 1.38 (1.13, 1.69) | 0.002 |
|  | Q3 (16-29) | 323 (13.5) | 297 (12.4) | 1.23 (1.00, 1.50) | 0.046 |
|  | Q4 (>29) | 342 (14.3) | 259 (10.8) | 1.61 (1.30, 1.98) | <0.001 |
|  |  |  |  |  |  |
| **Avg. daily lifetime g/ ethanol consumption^^^** | None / low | 1874 (77.0) | 1999 (81.0) | Reference |  |
|  | High (>24.6 g/d) | 560 (23.0) | 470 (19.0) | 1.27 (1.08, 1.50) | 0.005 |

**^*^Logistic regression adjusted for:** Sex, age, BMI, education level, history of colorectal cancer in first degree relative, previous endoscopy, diabetes, ever NSAIDs regular use and avg. daily lifetime alcohol consumption/ever smoking

**^+^Years since smoking cessation** were calculated from the last time the participant stopped smoking until diagnosis (cases) or interview (controls).

**^++^Pack-years** were calculated for smokers and former smokers as the number of years the participant smoked times the average number of cigarettes smoked per day. Reference group is never smokers.

^^^ High alcohol consumption was defined as the 4^th^ quartile of the average daily lifetime gram ethanol consumption among alcohol drinkers (> 24.6 g) and was compared in analyses to low/never consumption (≤ 24.6 g).
